# Improved organism detection in endophthalmitis: a comparison of traditional culture methods, pediatric blood culture bottles, and PCR

**DOI:** 10.1128/spectrum.00326-24

**Published:** 2024-04-22

**Authors:** A. L. Milligan, J. Soundrapandian, H. Petrushkin, N. Stone

**Affiliations:** 1 Emergency Department, Moorfields Eye Hospital NHS Foundation Trust, London, United Kingdom; 2 Pathology Department, Moorfields Eye Hospital NHS Foundation Trust, London, United Kingdom; 3 Uveitis Department, Moorfields Eye Hospital NHS Foundation Trust, London, United Kingdom; 4 Rheumatology Department, Great Ormond Street Hospital, London, United Kingdom; 5 Department of Clinical Microbiology, University College London Hospitals NHS Foundation Trust, London, United Kingdom; 6 Hospital for Tropical Diseases, University College London Hospitals NHS Foundation Trust, London, United Kingdom; University of Maryland School of Medicine, Baltimore, Maryland, USA

**Keywords:** blood culture bottle, infectious endophthalmitis, vitreous culture, PCR

## LETTER

Infectious endophthalmitis is a sight-threatening pathology. Early diagnosis and treatment are paramount to optimize visual outcomes. Organism identification is important to confirm diagnosis and guide antimicrobial therapy. Local microbiological data are valuable to guide empirical treatment. However, reported culture positivity rates vary significantly, from 22.5% to 36.5% for aqueous samples and 42.0% to 88.2% for vitreous samples (1).

Moorfields Eye Hospital is a tertiary ophthalmology referral center in London, UK. Samples are couriered to an external microbiology laboratory. A recent review of endophthalmitis cases revealed culture positivity in 21.7% of aqueous samples and 32.2% of vitreous samples (2). In efforts to guide best practice, two sequential diagnostic methods were evaluated.

**Fig 1:**
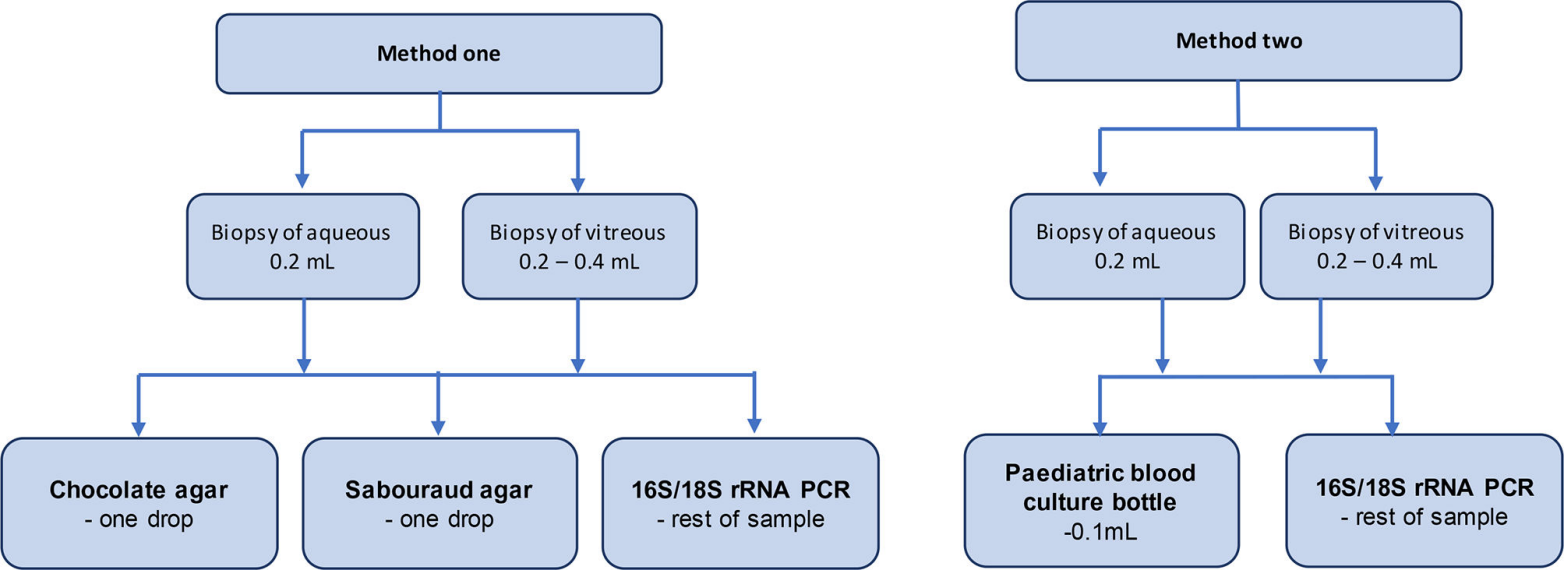
Flow diagram comparison of methods.

Inclusion criteria were new cases of suspected bacterial or fungal endophthalmitis in adults (***≥***16 years). Exclusion criteria were suspected viral etiology or alternative sampling methods, for example, vitrectomy. No patients had previous antibiotic treatment for endophthalmitis. A standard, sterile sampling technique was used, as described elsewhere (2). Aqueous (0.2 mL) and vitreous (0.2–0.4 mL) were processed separately. Method one employed traditional culture medium (TCM). One drop of fluid was plated on chocolate agar and Sabouraud’s dextrose agar, in the operating theatre, to minimize delay before receipt at the off-site laboratory. The plates were incubated at 37° Celsius prior to couriering. The remaining sample was sent for 16S (bacterial) and 18S (fungal) rRNA gene PCR analysis. In method 2, half of the sample was inoculated into an aerobic pediatric blood culture bottle (BCB) and placed in an automated blood culture detection system (BD Bactec, Beckton Dickinson, NJ). The remaining half had 16S/18S rRNA PCR performed (see Fig. 1).

TCM culture positivity was 14.3% (10.5% aqueous and 17.4% vitreous). There was a notable improvement when BCBs were introduced. Overall culture positivity increased to 37.8% (28.6% aqueous and 43.5% vitreous). 16S rRNA PCR was positive in 10.4% of samples (6.5% aqueous and 13.9% vitreous) (see Table 1). There were no positive 18S rRNA PCR results.

**TABLE 1:** Diagnostic yields of different techniques

	Sample type	Number of samples taken (%)	Number of positive samples (%)
Method 1 01/10/2021 until 01/10/2022	Aqueous culture solid plates	19/26 (73.0%)	2/19 (10.5%)
	Vitreous culture solid plates	23/26 (88.5%)	4/23 (17.4%)
	Total: culture solid plates	42/52 (80.8%)	6/42 (14.3%)
	Aqueous PCR	15/26 (57.7%)	1/15 (6.7%)
	Vitreous PCR	14/26 (53.8%)	1/14 (7.1%)
	Total: PCR	29/52 (55.8%)	2/29 (6.9%)
	Aqueous + vitreous culture	18/26 (69.2%)	1/18 (5.6%)
	Aqueous/vitreous culture + PCR	15/26 (57.7%)	0
Method 2 01/08/2023 until 10/12/2023	Aqueous blood culture bottles	14/27 (51.9%)	4/14 (28.6%)
	Vitreous blood culture bottles	23/27 (85.2%)	10/23 (43.5%)
	Total: blood culture bottles	37/54 (68.5%)	14/37 (37.8%)
	Aqueous PCR	16/27 (59.3%)	1/16 (6.3%)
	Vitreous PCR	22/27 (81.5%)	4/22 (18.2%)
	Total: PCR	38/54 (70.4%)	5/38 (13.2%)
	Aqueous + vitreous culture	12/27 (44.4%)	3/12 (25%)
	Aqueous/vitreous culture + PCR	26/27 (96.3%)	4/26 (15.4%)

The identified isolates were primarily coagulase-negative *Staphylococcus* (61.8%), and *Streptococcus* species (14.7%) (see Table 2). No fungi were isolated. In one case, 16S rRNA PCR identified *Moraxella nonliquefaciens* in an aqueous sample that was not identified by culture. Beyond this, rRNA PCR provided no additional information.

**TABLE 2:** Bacterial species identified

	Sample type (n)	Species identified	n	Source (n)
Method 1 01/10/2021 until 01/10/2022	Aqueous culture solid plates (2)	*Enterococcus gallinarum* *Staphylococcus warneri* *Staphylococcus epidermidis*	1 1 1	Post-cataract surgery
		*Haemophilus influenzae*	1	Bleb related
	Vitreous culture solid plates (4)	*Enterococcus gallinarum* *Micrococcus luteus* *Staphylococcus haemolyticus* *Staphylococcus capitis*	1 1 1 1	Post-cataract surgery
		*Streptococcus pneumoniae*	1	Post-intravitreal injection
		*Staphylococcus haemolyticus*	1	Post-vitrectomy
		*Staphylococcus hominis*	1	Post-intravitreal injection
	Aqueous PCR (1)	*Cutibacterium acnes*	1	Bleb related
	Vitreous PCR (1)	*Staphylococcus pasteuri* *Staphylococcus warneri*	1 1	Post-intravitreal injection
Method 2 01/08/2023 until 10/12/2023	Aqueous blood culture bottles (4)	*Staphylococcus epidermidis* *Staphylococcus haemolyticus*	1 1	Bleb related
		*Staphylococcus epidermidis*	1	Bleb related
		*Streptococcus gordonii*	1	Bleb related
		*Staphylococcus lugdenensis*	1	Bleb related
	Vitreous blood culture bottles (10)	*Staphylococcus epidermidis*	5	Post-intravitreal injection (4) Post-vitrectomy (1)
		*Streptococcus gordonii*	1	Bleb related
		*Staphylococcus lugdenensis*	1	Bleb related
		*Streptococcus pneumoniae*	1	Post-vitrectomy
		*Pseudomonas aeruginosa*	1	Post-cataract surgery
		*Bacillus mycoides*	1	Bleb related
	Aqueous PCR (1)	*Moraxella nonliquefaciens*	1	Bleb related
	Vitreous PCR (4)	*Staphylococcus epidemidis*	3	Post-intravitreal injection (3)
		*Streptococcus pneumoniae*	1	Post-vitrectomy
Total		*Staphylococcus species*	21/34 (61.8%)	
		*Streptococcus species*	5/34 (14.7%)	

Inoculation of BCBs has been advocated for the microbiological diagnosis of endophthalmitis for over 20 years. It is an established method in other sterile sites, for example, joint, pleural, or cerebrospinal fluid (3). Prior BCB studies have demonstrated a significantly higher sensitivity in organism detection, up to 64.7% (4–7). BCBs have been reported as having a favorable performance in fungal detection compared to TCM (8). They are particularly useful in settings with an off-site microbiology laboratory. Other benefits include ease of processing with reduced contamination, ease of storage and transport (room temperature rather than incubation), longer expiry, and faster results. This study has limitations in that it was performed in a single center with small numbers and without randomization. In addition, the clinical significance of isolates, in particular coagulase-negative staphylococci, can be difficult to prove; albeit this is a limitation in all similar studies. The observed difference has led to the universal introduction of BCBs in endophthalmitis diagnosis at our tertiary center.
